# Influence of Fluorine Doping on Rutile TiO_2_ Nanostructures for Visible-Light-Driven Photocatalysis: A DFT + U Study

**DOI:** 10.3390/nano15090694

**Published:** 2025-05-05

**Authors:** Fikadu Takele Geldasa, Francis Birhanu Dejene

**Affiliations:** Department of Chemical and Physical Sciences, Walter Sisulu University, Private Bag X1, Mthatha 5117, South Africa; fdejene@wsu.ac.za

**Keywords:** semiconductor, photocatalysis, renewable energy, bandgap, pollutant degradation

## Abstract

In this work, a density functional theory (DFT) with Hubbard correction (U) approaches implemented through the Quantum ESPRESSO code is utilized to investigate the effects of fluorine (F) doping on the structural, electronic, and optical properties of rutile TiO_2_. Rutile TiO_2_ is a promising material for renewable energy production and environmental remediation, but its wide bandgap limits its application to the UV spectrum, which is narrow and expensive. To extend the absorption edge of TiO_2_ into the visible light range, different concentrations of F were substituted at oxygen atom sites. The structural analysis reveals that the lattice constants and bond lengths of TiO_2_ increased with F concentrations. Ab initio molecular dynamics simulations (AIMD) at 1000 K confirm that both pristine and F-doped rutile TiO_2_ maintains structural integrity, indicating excellent thermal stability essential for high-temperature photocatalytic applications. Band structure calculations show that pure rutile TiO_2_ has a bandgap of 3.0 eV, which increases as the F concentration rises, with the 0.25 F-doped structures exhibiting an even larger bandgap, preventing it from responding to visible light. The absorption edge of doped TiO_2_ shifts towards the visible region, as shown by the imaginary part of the dielectric function. This research provides valuable insights for experimentalists, helping them understand how varying F concentrations influence the properties of rutile TiO_2_ for photocatalytic applications.

## 1. Introduction

Nowadays, different materials including semiconductors have gained significant attention for addressing renewable energy challenges and environmental remediation. Photocatalysis is a green and sustainable technology that utilizes light and catalysts to drive a variety of chemical processes, offering promising solutions for applications such as solar energy conversion, environmental remediation, and hydrogen production. The efficiency of semiconductors used as photocatalysts is fundamentally governed by the behavior of photoinduced charge carriers [1,2]. Upon light absorption, electron–hole pairs are generated, and their subsequent separation, migration to reactive sites, and suppression of recombination are critical to achieving high photocatalytic performance. The dynamics of photoinduced charge carriers, including their lifetimes, recombination rates, and diffusion pathways, are highly sensitive to the electronic structure of the semiconducting material [3,4,5]. A key requirement for any semiconducting material used as a photocatalyst is its ability to effectively harvest light, particularly in the visible range of the solar spectrum.

Rutile TiO_2_ has become a key semiconducting material in photocatalytic applications for renewable energy and environmental remediation due to its outstanding stability, non-toxicity, and strong photocatalytic activity under UV irradiation [6,7,8]. However, the inherent wide bandgap of approximately 3.0 eV limits its efficacy under visible light [9], which constitutes a significant portion of the solar spectrum. Consequently, the modification of TiO_2_ to enhance its optical properties and photocatalytic performance under visible light has become a focal point of research in material science. Dye sensitization is among the methods used to overcome the wide bandgap limitation of TiO_2_, expanding its utility in photocatalytic and optoelectronic applications under visible light irradiation. In this strategy, light-absorbing dye molecules are anchored to the TiO_2_ surface, where they serve as visible-light harvesters. Upon excitation by visible light, these dyes can inject electrons into the conduction band of TiO_2_, initiating charge separation and enabling photocatalytic activity under visible light, as in dye-sensitized solar cells [10,11,12].

Among other modifications, doping metal and non-metal elements has attracted researchers’ attention. Thus, the bandgap of TiO_2_ can be reduced via doping with other elements, which can make it highly active under visible lights [13]. Fluorine (F) doping is a promising approach for tailoring the electronic structure of TiO_2_ [14]. The introduction of F atoms into the TiO_2_ lattice is anticipated to influence its bandgap and enhance its photocatalytic efficiency by facilitating charge carrier generation and separation [15].

Previous theoretical and experimental studies have suggested that F doping can lead to an increase in visible light absorption and a reduction in recombination rates, ultimately improving the photocatalytic activity of TiO_2_ polymorphs. For instance, Tosoni et al. [16] studied the effect of F doping on the structure of bulk rutile, anatase, and brookite phases of TiO_2_ by employing density functional theory (DFT). Their finding shows that F doping does not lead to the reduction of bandgap in rutile and brookite; however, it shows remarkable bandgap reduction in the anatase TiO_2_, which is convenient for photocatalytic purposes under visible light irradiation. Miyoshi et al. [17] also utilized DFT to investigate the effect of nitrogen/fluorine co-doping on the photocatalytic activity of rutile TiO_2_. Salehi-Aba et al. [18] investigated the effect of F and N doping on the electronic properties of rutile TiO_2_ quantum dots using DFT calculations. The results indicated that F doping increased the absorption efficiency compared to N doping, which was attributed to the reduction of auger recombination. Zhao et al. [19] fabricated F-doped TiO_2_ for photocatalysis application under visible light. According to their results, the presence of F reduced the bandgap, altered the electronic structure, and enabled the photon absorption edges to shift to a longer wavelength. Lin et al. [20] synthesized F-doped TiO_2_ loaded with Ag using the sol–gel method for wide-range absorption of light for photocatalytic degradation. Todorova et al. [21] also synthesized nanocrystalline F-doped rutile TiO_2_ using a sol–gel route for photocatalytic application of acetone decomposition with the shifted absorption edge of F-doped to visible light. Díez et al. [22] synthesized F-doped TiO_2_ using one-step synthesis and utilized it for the degradation of toxic and stable pollutants (methylene blue and bisphenol). Fittipaldi et al. [23] developed boron and fluorine co-doped rutile TiO_2_, achieving enhanced photocatalytic activity for the decolorization of methyl orange under visible light exposure.

The influence of fluorine doping on other material systems, such as graphene-like structures CF_x_ and sulfur–fluorine co-doped carbon thin films CS_x_F_y_, has also been investigated in previous studies [24,25]. These works employed efficient DFT simulations within the framework of the Synthetic Growth Concept (SGC) to gain insight into the evolution of structural and bonding patterns during material formation. By correlating the growth environment with the resulting atomic configurations and electronic properties, such studies have provided valuable guidance for the controlled fabrication and property tuning of F-doped nanomaterials. This approach highlights the importance of theoretical modeling in supporting experimental synthesis and underscores the relevance of applying similar strategies to F-doped rutile TiO_2_ systems.

Despite considerable research highlighting the influence of dopants on the structural, electronic, and optical properties of rutile TiO_2_, there is still limited work focused specifically on rutile TiO_2_ compared to the anatase TiO_2_ phase. To the best of our knowledge, no DFT + U studies have been conducted on F-doped rutile TiO_2_ with varying F concentrations to comprehensively investigate the influence of F doping on its structural, electronic, and optical properties for photocatalysis applications under visible light.

In this study, we employ DFT coupled with Hubbard U correction (DFT + U) to thoroughly investigate the effects of F doping on the structural, electronic, and optical properties of rutile TiO_2_. This computational framework enables a more accurate description of the electronic interactions and correlations in strongly correlated systems, taking into account the localized nature of the 3d orbitals of Ti. By systematically varying the concentration of F dopants, we aim to elucidate the optimal doping strategy that maximizes the photocatalytic potential of rutile TiO_2_. The significance of this paper lies not only in understanding the fundamental modifications induced by F doping but also in paving the way for the design of high-performance photocatalysts capable of harnessing solar energy more efficiently.

## 2. Computational Details

In this study, we performed density functional theory (DFT) calculations using Quantum ESPRESSO [26] to investigate the effects of F doping on the structural, electronic, and optical properties of rutile TiO_2_ for photocatalytic applications. The DFT calculations employed the generalized gradient approximation (GGA) with the Perdew–Burke–Ernzerhof (PBE) functional [27]. Initially, a self-consistent field input file was constructed using experimentally reported crystallographic data for rutile TiO_2_, with lattice parameters set to a = b = 4.5998 Å, c = 2.9592 Å, volume = 62.6125, and α = β = γ = 90° [28]. Crystal structure modeling and visualization were conducted using XCrySDen [29] and VESTA [30] software. The projector augmented wave (PAW) pseudopotential type was utilized to describe the electrons and ionic core interactions. The valence electron configurations for Ti (3d^2^ 4s^2^), O (2s^2^ 2p^4^), and fluorine (2s^2^ 2p^5^) were considered. The cutoff energy and k-point convergence tests were conducted, yielding a converged cutoff energy of 46 Ry and Monkhorst–Pack k-points of 6×6×7 for the calculations. The convergence criteria for total energy and forces were set to 10⁻^6^ eV and 10⁻^3^ eV/Å, respectively. To address the underestimation of the bandgap in transition metal oxides using standard GGA, we incorporated a Hubbard U correction to account for the self-interaction of localized Ti 3d orbitals. The optimized U value derived from our previous publication, which was 7.5 eV [31], which provided improved alignment with experimental bandgap values, is used in the current study. In our previous work, the Hubbard U parameter was systematically optimized using DFT calculations to reproduce the experimental bandgap of rutile TiO_2_. The U value varied from 0 to 10.5 eV. At U = 0 eV, the calculated bandgap was 1.86 eV. As U increased, the bandgap also increased, reaching a maximum value of 3.03 eV at U = 7.5 eV. Beyond this point, further increases in U resulted in a decrease in the bandgap. Thus, the choice of U = 7.5 eV is justified, as it yields a bandgap in excellent agreement with the experimental measurements. A 2 × 2 × 1 supercell was constructed to analyze the effect of F doping by varying the concentrations of F as 0.0625 named F1, 0.125 named F2, 0.188 named F3, 0.25 named F4 substituted at oxygen sites. For band structure calculations, high-symmetry k-points were employed, while a denser k-point grid of 16 × 16 × 20 facilitated total and projected density of states (TDOS and PDOS) analyses. Optical properties, including the real and imaginary parts of the dielectric functions, were computed using the Kramers–Kronig relationship and momentum matrix elements of unoccupied and occupied states, respectively [32].

In order to comprehensively understand the influence of F doping on rutile TiO_2_, we also considered different possible defect configurations. Specifically, we modeled the highest concentration (0.25) of F substitution at different O lattice sites within the TiO_2_ lattice. These configurations were chosen based on their potential impact on the electronic and structural properties of the material. All defect configurations were constructed within a 2 × 2 × 1 supercell by changing different F dopant arrangement within the rutile TiO_2_ lattice. The formation energies of each defect configuration were calculated under titanium-rich conditions to assess their relative thermodynamic stability. The comparative analysis of these configurations provides insight into the most energetically favorable doping mechanism and its effect on photocatalytic behavior under visible light.

## 3. Results and Discussion

### 3.1. Structural Analysis

**Figure 1:**
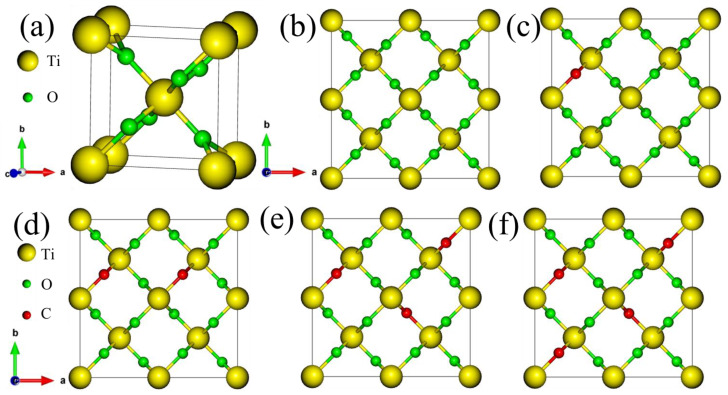
Crystal structures of (**a**) bulk rutile TiO_2_, (**b**) 2 × 2 × 1 supercell of pure rutile TiO_2_, and Ti_8_O_16-x_F_x_ with doping concentrations of (**c**) 0.025, (**d**) 0.125, (**e**) 0.188, and (**f**) 0.25.

Crystallinity of materials has a significant effect on material properties. Rutile TiO_2_ possesses a tetragonal crystal structure with space group P4_2_/mnm (No. 136) [33], as depicted in Figure 1a. The Ti atoms occupy the 2a Wyckoff positions, with fractional coordinates (0, 0, 0) and (1/2, 1/2, 1/2). The O atoms reside at the 4f Wyckoff positions, with coordinates (±*u*, ±*u*, 0) and ± (*u* + 1/2, 1/2 − *u*, 1/2), where *u* = 0.30493. Six of the O^2−^ atoms are bonded with a single Ti^4+^ atom found at the center position in the crystal structure, forming a Ti-O chemical bond. The constructed structure of a 2 × 2 × 1 supercell having 24 atoms (Ti_8_O_16_) is shown in Figure 1b. This supercell is built to identify the effect of different F concentration doping on the properties of rutile TiO_2_. The structures of Ti_8_O_16_ doped with different F concentrations are shown in Figure 1c–f. The position of the dopant is selected based on the atomic positions in the supercell. For instance, in the 0.0625 doping, a single O atom at positions 0.15229, 0.65229, and 0.0000 is removed and replaced by F.

**Figure 2:**
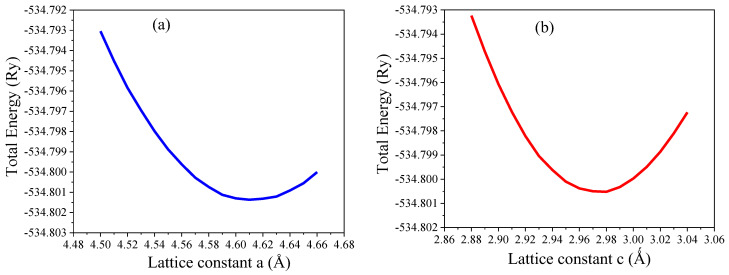
(**a**) The computed lattice constant *a* and (**b**) lattice constant *c* under equilibrium conditions.

**Figure 3:**
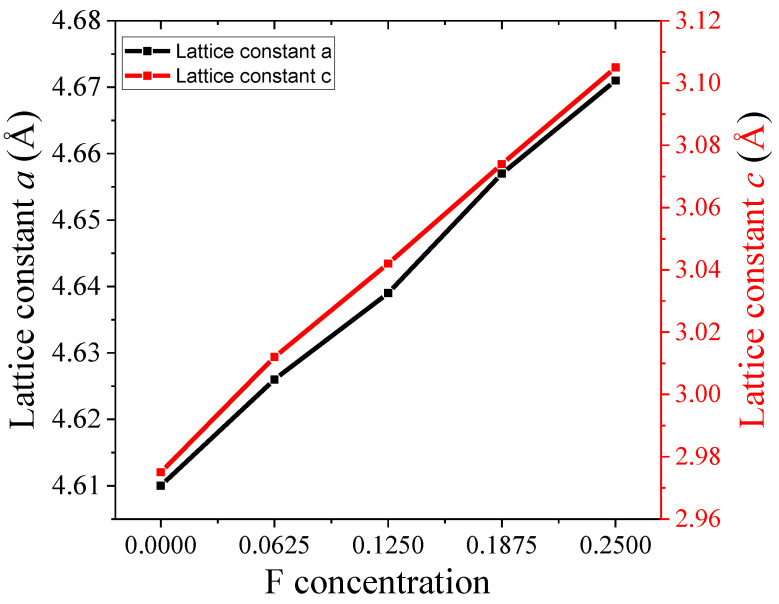
Variation in lattice constants *a* and *c* with F concentrations.

The computed lattice constants for pure rutile TiO_2_ at minimum total energy are presented in Figure 2. As presented in Figure 2a, the lattice constant obtained for both *a* and *b* is 4.610 Å, and is 2.975 Å for c, as shown in Figure 2b, which agrees well with the experimental results [34]. The lattice constants obtained for pure and F-doped rutile TiO_2_ after full geometry optimizations are given in Table 1. Additionally, other DFT reports and experimental results are included in Table 1 to compare the present work with other studies. The lattice constants obtained for pure rutile TiO_2_ agrees well with the reported values, except for small deflections in some DFT reports. When concentrations of F increased, the lattice constant also increased, as shown in Figure 3, which may be attributed to the atomic radius difference of fluorine and oxygen [35]. Thus, the lattice constants of crystal increased as the concentration of F atoms increased. This is because when the number of dopant atoms increases, the accommodation of foreign atoms within the crystal lattice increases, which results in expansion of the crystal lattice.

Variations in the dopant configuration may also contribute to the observed increase in lattice constants with higher fluorine doping concentrations. The local atomic environment surrounding the dopant plays a crucial role in determining the extent of lattice distortion. When F atoms substitute for O atoms in close proximity, forming clustered configurations, they can induce significant local strain, resulting in greater distortions in Ti–O/F bond lengths and bond angles.

The obtained bond angles and bond lengths of pure and doped TiO_2_ is presented in Table 2. As shown in Table 2, bond length increased as concentrations of F increased, which can be related to the distortion of lattice due to increasing dopant concentrations [36].

### 3.2. Formation Energy

The formation energy of F-doped rutile TiO_2_ represents the energy required to introduce the F atom into the host material, rutile TiO_2_. The general expression for the formation energy of F-doped TiO_2_ is given by [36]

(1) $$E_{f}=E_{total}\left(F\;doped\;TiO_{2}\right)-E_{total}\left(pure\;TiO_{2}\right)-n(\mu_{F}-\mu_{O})$$

where

- $E_{f}$ is the formation energy of the F-doped TiO_2_;
- $E_{total}\left(F\;doped\;TiO_{2}\right)$ and $E_{total}\left(pure\;TiO_{2}\right)$ are the total energies of the F-doped TiO_2_ and pure TiO_2_, respectively;
- $\mu_{F}$ and $\mu_{O}$ are the chemical potentials of F and O, respectively;
- n is the number of oxygen atoms substituted by F.
- The chemical potentials follow the relationship

(2) $$\mu_{{TiO}_{2}}={2\mu }_{O}+\mu_{Ti}$$

where $\mu_{O}$ is half the total energy of an oxygen molecule and the energy of a titanium atom in the bulk TiO_2_. The chemical potential of the dopant corresponds to the total energy of an isolated dopant atom within its unit cell [38]. The formation energy is calculated under titanium-rich conditions, where F substitutes for O by eliminating a single oxygen atom. As shown in Table 2, all F concentrations doped into rutile TiO_2_ exhibit negative formation energies, indicating that F is energetically favorable in the rutile TiO_2_ structure [39]. Among the F concentrations, the 0.25 doping possessed greater negative formation energy than other concentrations, which indicates the easiest concentration to incorporate into rutile TiO_2_ lattices.

**Figure 4:**
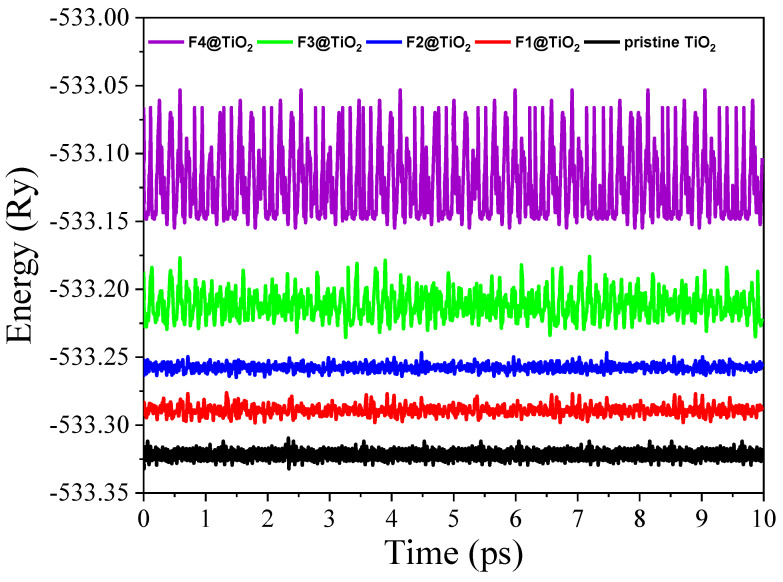
Ab initio finite temperature molecular dynamics for pristine rutile TiO_2_ and TiO_2_ doped with various F concentrations.

Furthermore, in order to evaluate the thermal stability of pristine TiO_2_ and TiO_2_ doped with various F concentrations, we performed ab initio finite temperature molecular dynamics simulations. These simulations were conducted over a period of 10 picoseconds (ps) with 1 femtosecond (fs) time steps at a temperature of 1000 K. The resulting trajectories depicted in Figure 4 provide crucial insights into the structural integrity of the TiO_2_ system under high-temperature conditions. As shown in Figure 4, the structural stability of pristine TiO_2_ is maintained even at 1000 K, with no significant deviations in the atomic positions or the overall lattice structure, suggesting that rutile TiO_2_ is thermally stable at this temperature. This stability is crucial for photocatalytic applications of rutile TiO_2_ where high-temperature conditions are often encountered. For the F-doped TiO_2_, the energy trajectories also exhibit a relatively steady trend over time, confirming that the material remains stable at elevated temperatures. While the energy profiles for the F-doped materials show slight variations as the F concentration increases, these variations are not significant enough to indicate any structural collapse or major disruption of the lattice. The energy remains relatively stable throughout the simulation period, suggesting that even at higher doping concentrations, the F incorporation does not adversely affect the thermal stability of the rutile TiO_2_ structure.

### 3.3. Electronic Properties

#### 3.3.1. Electronic Band Structure

**Figure 5:**
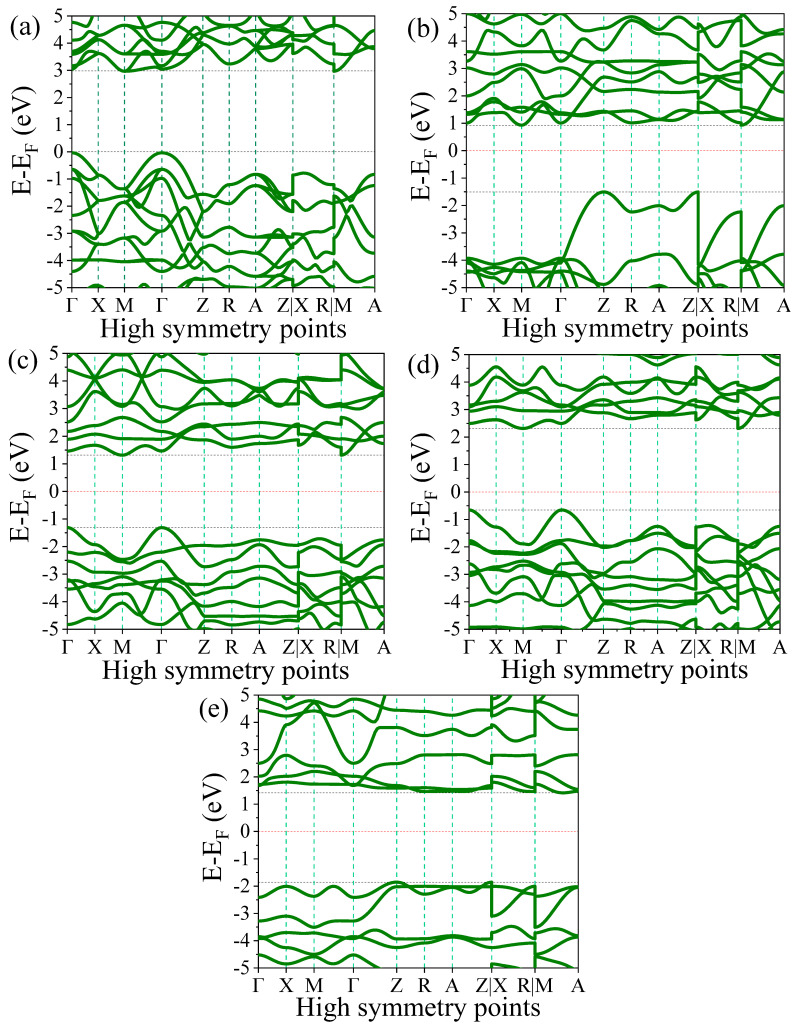
Band structures of (**a**) pure rutile TiO_2_ and TiO_2_ doped with concentrations of (**b**) 0.0625, (**c**) 0.125, (**d**) 0.1875, and (**e**) 0.25.

To calculate the electronic band structure, we used the high-symmetry k-points Γ—X—M—Γ—Z—R—A—Z|X—R|M—A of reduced Brillioun zone. The electronic band structures of pure and F-doped TiO_2_ with various concentrations are computed using DFT + U, as shown Figure 5a–e. In the figures, the zero energy indicates the Fermi energy levels (E_F_). For the electronic band structure of pure rutile TiO_2_, it was found that the conduction band minimum (CBM) is located at the M symmetry point with a magnitude of 2.1365 eV and the valence band maximum (VBM) is located at Γ symmetry point with magnitude −0.865 eV, as shown in Figure 5a. From the band structure computation, the bandgap of pure TiO_2_ was shown to be indirect, with a 3.0 eV value, as shown in Table 3. The obtained bandgap value of pure TiO_2_ matches well with previously reported works. In our previous work, we obtained a similar indirect bandgap with a magnitude of 3.03 eV value using DFT + U and 1.86 eV without Hubbard correction [31].

The incorporation of F impurities in rutile TiO_2_ at concentrations of 0.0625, 0.125, 0.1875, and 0.25 caused a significant change in the electronic band structure of TiO_2_. Table 3 shows the computed bandgap of the values of doped materials at various concentrations. For the 0.0625-doped TiO_2_, the CBM is located at the M high-symmetry point with a minimum energy of 0.9297 eV and the VBM is located at the Z high-symmetry point with a maximum energy of −1.506 eV, as shown in Figure 5b. The CBM is located at the same symmetry point as pure TiO_2_. However, the VBM location is shifted to Z high-symmetry points. The obtained bandgap is indirect, with a magnitude of 2.44 eV, which showed 0.56 eV reductions compared to pure TiO_2_.

The obtained indirect bandgap of 2.63 eV at 0.125 and 2.97 eV at 0.1875 concentrations are shown in Figure 5c and 5d, respectively. Similar to that of pure TiO_2_, the CBM of these doped materials is located at M high-symmetry points with the minimum energy values of 1.3147 eV and 2.3135 eV, and the CBM is located at Γ high-symmetry points with the maximum energy values of −1.3128 eV and −0.6551 eV for the 0.125 and 0.1875 concentration-doped materials, respectively. For the F-doped TiO_2_ with concentrations of 0.125 and 0.1875, the bandgap narrowed by 0.37 eV and 0.03 eV, respectively, compared to pure TiO_2_.

Unlike other concentrations, the 0.25-doped materials showed an increased bandgap compared to pure TiO_2_. As shown in Figure 5e, its CBM is found at R and A high-symmetry points with a minimum energy of 1.413 eV, and its VBM is found at Z high-symmetry point with a maximum energy of −1.8525 eV, which resulted in an indirect bandgap with a magnitude value of 3.27 eV. This bandgap value exceeds that of pure TiO_2_ by 0.27 eV. In general, with an increase in F doping concentrations, the bandgap value increased [40]. Specifically, for the 0.25 F doping concentration, the bandgap value exceeds that of the host material due to the Burstein–Moss effect [41,42,43]. Thus, when a semiconductor is doped, the introduction of dopant atoms fills the energy states near the conduction band. This shift in the electron distribution effectively raises the energy of the conduction band edge, leading to an apparent widening of the bandgap as the absorption edge moves to higher energies. Furthermore, the bandgap widening can be attributed to the high electronegativity of fluorine, which affects the electronic structure by reducing the density of states near the Fermi level and increasing the energy separation between the valence and conduction bands. This leads to a systematic increase in the band gap as the fluorine content increases.

In other F doping concentrations, the bandgap values were narrower than that of pristine TiO_2_, which can result in shifting of the absorption edge of TiO_2_ to the visible light for photocatalysis applications under visible light irradiation. On the other hand, the indirect bandgap properties of the doped materials can reduce the recombination of photoinduced electrons and holes because electrons that are excited to the conduction bands must travel across k-space before the recombination of holes in the valence bands [44,45]. Thus, the reduction of photoinduced charge carrier recombination improves the photocatalytic activity of TiO_2_.

At higher F doping concentrations, the relative positions of dopant atoms within the TiO_2_ lattice can have a pronounced effect on the resulting electronic structure and, consequently, on the materials’ photocatalytic performance. While our study has provided insights into the overall electronic modifications induced by F doping, it is important to acknowledge that dopant configurations, particularly the spatial distribution of F atoms, can introduce further complexity.

**Figure 6:**
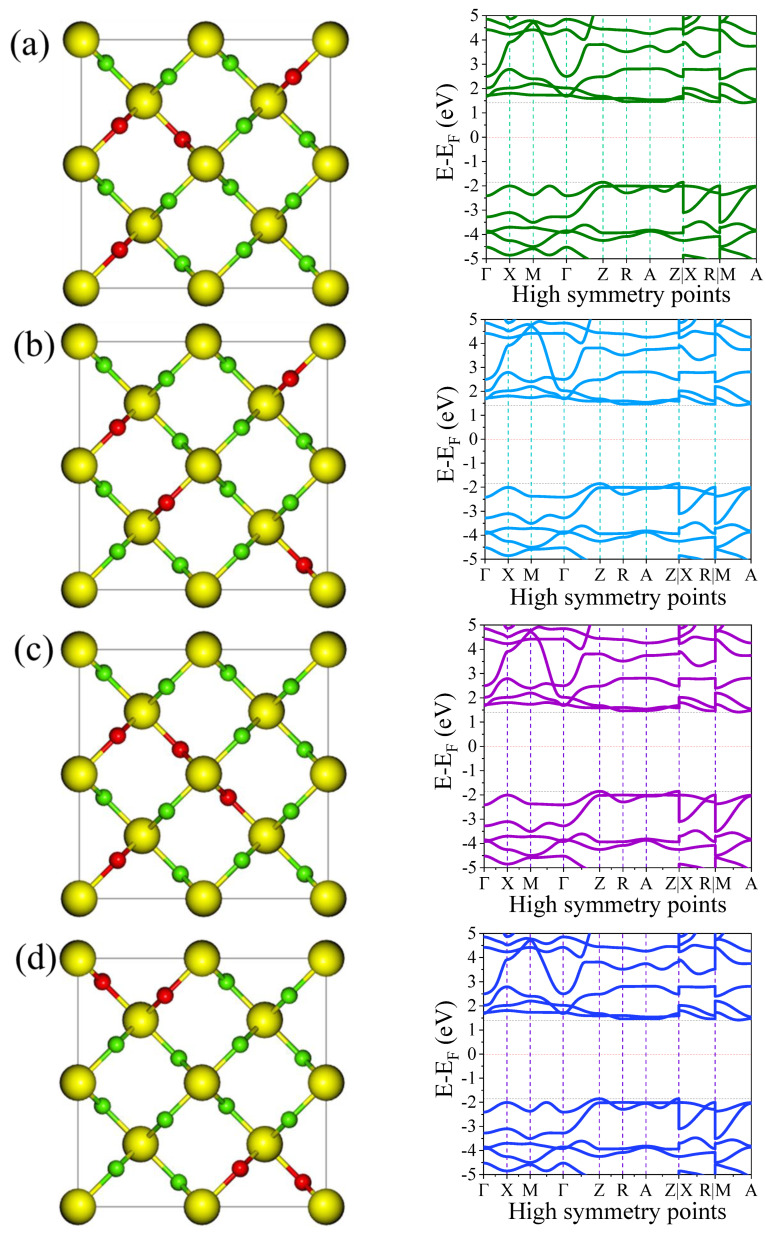
Band structures of the 0.25 F-doped rutile TiO_2_ for different dopant arrangements in TiO_2_ lattice.

To assess the impact of different relative arrangements of defects on the band structure at the highest dopant concentration, we performed a set of test DFT calculations. The left Figure 6a–d shows different relative arrangements of dopants. As shown in the right Figure 6a–d, the calculated band structures for various relative arrangements of dopant atoms at the highest concentration exhibit only minor variations. Specifically, the bandgap values remain nearly unchanged, indicating that the spatial distribution of defects has a minimal influence on key electronic properties such as the VBM, CBM, and overall bandgap. These values are summarized in Table 4, which confirms the small numerical differences across the different configurations. The observed bandgap fluctuations are within 0.0013 eV, which is negligible in the context of DFT calculations and falls well within typical numerical tolerances. While subtle changes in the dispersion of the valence and conduction bands can be noted, primarily affecting the curvature near the band edges, these do not significantly alter the electronic transitions or the effective bandgap. Such behavior can be attributed to the localized nature of the defect-induced states and the relatively uniform electronic screening in the host lattice, which reduces the impact of dopant spatial configuration.

Furthermore, we calculated the formation energies for the various F defect configurations considered in this study to investigate their thermodynamic stability. As shown in Table 4, the computed formation energies are −3.68 eV, −3.57 eV, −3.52 eV, and −3.73 eV for the arrangement in Figure 6a–d, respectively. These results indicate that the defect configuration with a formation energy of −3.73 eV is the most energetically favorable, while the other configurations are slightly less stable, with energy differences of up to 0.21 eV. These relatively small differences suggest that several configurations could be present, depending on the modeling conditions.

**Table 3:** The computed bandgap of pure rutile TiO_2_ and TiO_2_ doped with various F concentrations.

Materials	VBM (eV)	CBM (eV)	Bandgap (eV)	Other DFT +U Bandgap (eV)	Experimental Bandgap (eV)
Pure TiO_2_	−0.8650	2.1365	3.00	3.03 ^a^	3.00 ^b^
F1@TiO_2_	−1.5060	0.9297	2.44	---	---
F2@TiO_2_	−1.3128	1.3147	2.63	---	---
F3@TiO_2_	−0.6551	2.3135	2.97	---	---
F4@TiO_2_	−1.8525	1.4130	3.27	---	---

Reported values are taken from Ref. ^a^ [46] and ^b^ [47].

**Table 4:** The computed bandgap, VBM, and CBM of different dopant arrangements in TiO_2_ lattice.

Dopant Arrangement	VBM (eV)	CBM (eV)	Bandgap (eV)	Formation Energy (eV)
a	–1.8525	1.4130	3.2655	−3.68
b	–1.8522	1.4120	3.2642	−3.57
c	–1.8521	1.4127	3.2648	−3.52
d	–1.8521	1.4124	3.2645	−3.73

#### 3.3.2. Total and Partial Density of States

**Figure 7:**
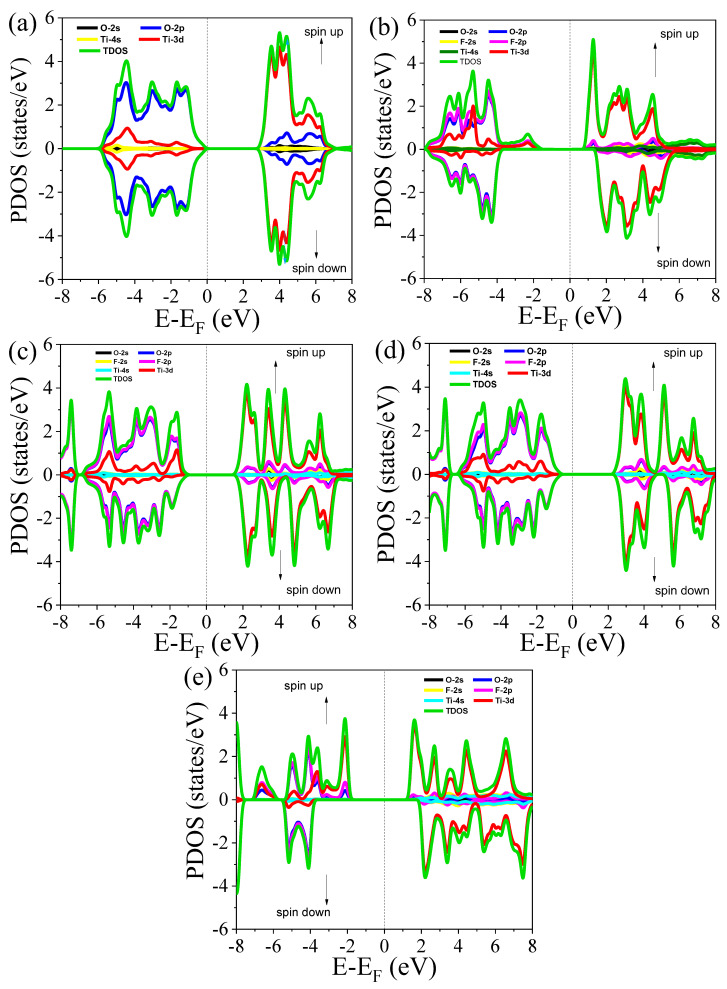
The TDOS and PDOS of (**a**) pure rutile TiO_2_ and TiO_2_ doped with concentrations of (**b**) 0.0625, (**c**) 0.125, (**d**) 0.1875, and (**e**) 0.25.

Total density of states (TDOS) represents the total electron states available at a given energy level for the entire system, and partial density of states (PDOS) breaks down the TDOS by specific atomic orbitals, showing contributions from different types of orbitals. To understand the dispersion of each orbital in pure and doped TiO_2_ crystal and its energy band structure, the TDOS and PDOS of Ti, O, and F were determined using the DFT + U approach [48]. The spin-polarized TDOS and PDOS of pure and F-doped rutile TiO_2_ is shown in Figure 6a–e using the photon energy from −8 eV to 6 eV. As shown in Figure 7a, it was discovered that the conduction band mainly originated from the contribution of the Ti 3d orbital and the minor contribution of the O 2p orbital for pristine TiO_2_. The O 2p orbital has a major contribution in the valence band of TiO_2_, with a minor contribution of the Ti 3d orbital. Thus, in the conduction band, the dominant peak originates from the influence of the Ti 3d orbital, and in the valence band, the dominant peak originates from the contribution of the O 2p orbital, indicating that the O 2p states are occupied states and the Ti 3d states are unoccupied. The spin-up and spin-down peaks of the TDOS positioned at the same energy (symmetric) for both the valence band and conduction band indicate that TiO_2_ is a non-magnetic material.

In the case of 0.0625-doped TiO_2_, conduction band originated from the major contribution of the Ti 3d orbital and minor contribution of the O 2p and F 2p orbitals, as shown in Figure 7b. The valence band is made up of the major contribution of the F 2p and O 2p orbitals. Unlike pristine TiO_2_, the position of the highest peaks for spin-up and spin-down is not located at the same energy (asymmetric). In the conduction band, the highest peak at the edge is 1.283 eV for spin-up and at 2.0 eV for spin-down. Specifically, the states found around −2.32 eV did not occur for spin-down polarization. Thus, F doping contributes to TiO_2_ acquiring magnetic properties, and the F 2p state is the main source of magnetization. In addition to these impacts, F-doped TiO_2_ possessed a higher density of states than pristine TiO_2_. In other doped materials, the F 2p orbital has an impact on the valence band similarly. Similar to pristine TiO_2_, Ti 3d contributes to the formation of a conduction band. When the F concentrations increased, the number of emerged peaks increased due to the increase in the defects. In all doped materials, the peak positions in the spin-up and spin-down are not located at the same energy and are not symmetric, which indicates that F doping contributes to TiO_2_ exhibiting magnetic behavior. Furthermore, the induced magnetic behavior of F-doped rutile TiO_2_ improves the efficiency of absorbing visible light since the magnetic materials have shown high potential to absorb significant visible lights [49]. On the other hand, this property of materials enhances the photocatalytic activity of TiO_2_ in visible light irradiation.

**Figure 8:**
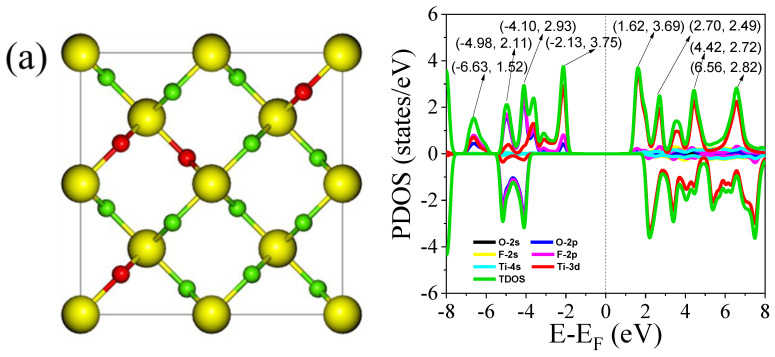

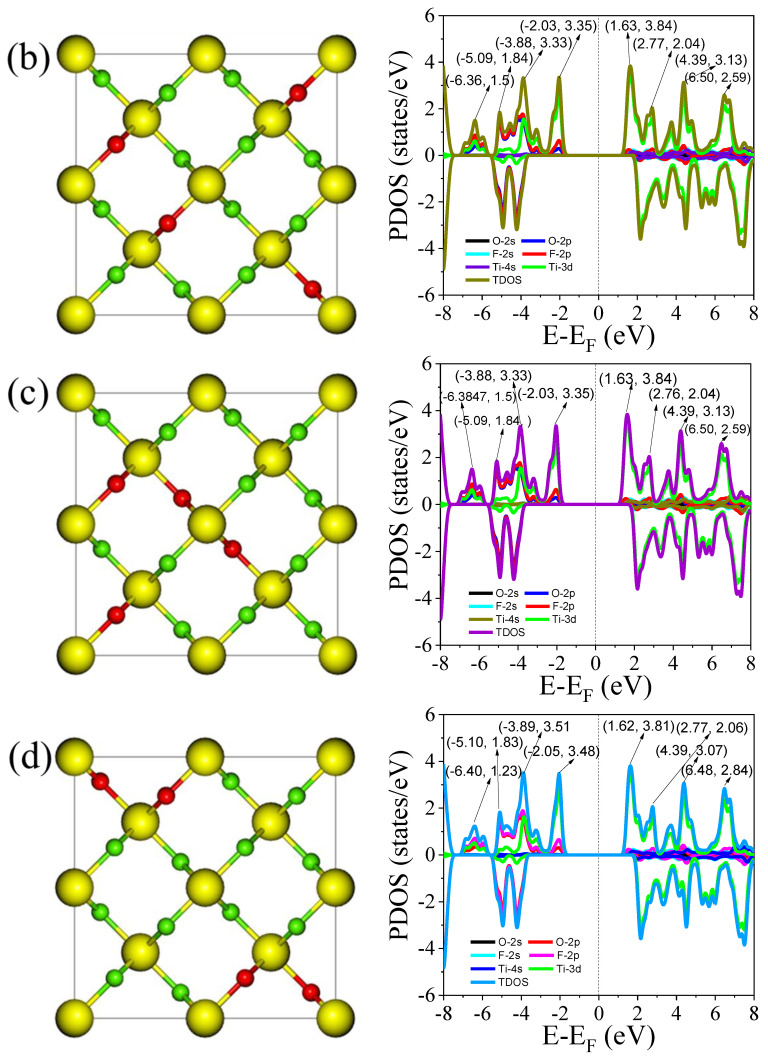
PDOS and TDOS of the 0.25 F-doped rutile TiO_2_ for different dopant arrangements in TiO_2_ lattice.

Furthermore, the influence of different dopant configurations, particularly at the highest dopant concentration, on the material’s density of states (DOS) was investigated. The calculated PDOS and TDOS for various dopant arrangements are presented in the right side Figure 8a–d. The left Figure 8a–d shows the different dopants arrangements in the TiO_2_ structure. Consistent with the band structure results, the spatial distribution of the dopant atoms has a minimal impact on the positions of the valence band maximum and conduction band minimum. Moreover, the spatial configuration of the dopants introduces only subtle changes in the TDOS profiles. As illustrated in Figure 8, the peak intensities of the TDOS may increase or decrease slightly depending on the specific dopant arrangement, but the overall variations are marginal. The positions of these peaks also exhibited very minor shifts across different configurations. These minimal variations in both intensity and peak positions can be attributed to the similar ionic radii of F and O. Since F and O are chemically and structurally compatible, substituting one for the other leads to negligible distortion of the electronic structure, thereby preserving the overall density of states profile. This suggests that the system exhibits a degree of defect tolerance with respect to dopant distribution, which could be advantageous for electronic and optoelectronic applications.

### 3.4. Charge Density

**Figure 9:**
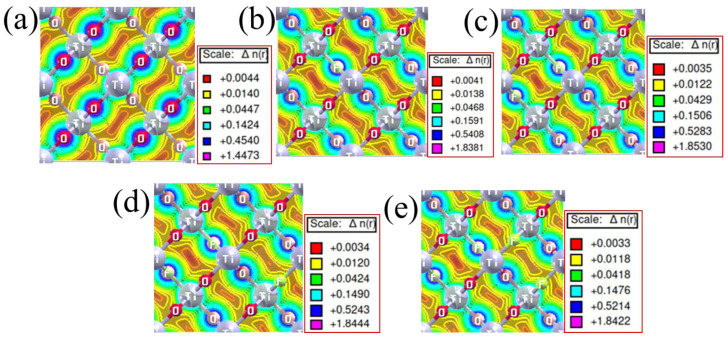
Charge density distribution in (**a**) pristine rutile TiO_2_ and (**b**) 0.0625, (**c**) 0.125, (**d**) 0.1875, and (**e**) 0.25 F-doped TiO_2_.

The investigation into the charge sharing and bonding nature of pristine rutile TiO_2_ and TiO_2_ materials doped with various F concentrations was carried out by computing the charge densities using self-consistent field calculations. The results, illustrated in Figure 9a–e, reveal significant insights into the structure and bonding characteristics of the material. In pristine rutile TiO_2_, the charge density is predominantly localized at the oxygen sites, as indicated by the color scale in Figure 9a. This suggests that the Ti-O bond is primarily ionic, with a strong electron density surrounding the oxygen atoms. The observed electron density distribution is consistent with the well-known ionic nature of TiO_2_, where oxygen atoms are more electronegative and attract electron density from titanium, leading to a strong Ti-O bond. Upon F incorporation, the charge density increases compared to the pristine material. The charge density map of the doped systems shows that the maximum charge density is now localized at the F sites, particularly for the 0.25 doping concentrations. This indicates a shift in the bonding nature, with the Ti-F bond becoming more ionic as F, which is highly electronegative, interacts with Ti atoms. The increased charge density at F sites suggests that the Ti-F interaction is stronger, likely due to the electron-withdrawing properties of F.

Among the different F doping concentrations, the system with 0.0625 F concentration exhibits the highest charge density, indicating that both Ti-O and Ti-F bonds are particularly strong in this material. This is likely due to an optimal amount of F incorporation, where the Ti-F bonding strength is maximized without creating an excessive number of defects or disrupting the lattice structure too much. At higher F doping concentrations (0.25), the charge density still shows an increase compared to the pristine material, but the maximum charge density is lower than in the 0.0625 F-doped case. This suggests that while Ti-O and Ti-F bonds remain strong, the bonding interactions might be less pronounced at lower doping levels. The relatively lower charge density at the F site could indicate that fluorine is less effectively incorporated, leading to weaker Ti-F interactions. The charge density analysis aligns with the partial density of states (PDOS) shown in Figure 7, where the overlap between the Ti (3d), O (2p), and F (2p) orbitals further corroborates the shift in bonding character upon F doping. The hybridization of Ti (3d) and O (2p) orbitals in pristine TiO_2_ indicates strong ionic Ti-O bonding, while the inclusion of F leads to an additional hybridization involving F (2p) orbitals, reflecting the stronger bonding character of the Ti-F bond.

### 3.5. Optical Properties

The optical properties of materials, including absorption, emission, reflection, and transmission, provide crucial information regarding materials’ behaviors that can be used in optoelectronic applications by varying photon energies. The physical quantities used to determine optical characteristics are dielectric constant $\varepsilon \left(\omega \right)$, absorption coefficient $\alpha \left(\omega \right)$, refractive index $n\left(\omega \right)$, reflectivity $R\left(\omega \right)$, extinction coefficient$\;\kappa \left(\omega \right)$, optical conductivity $\sigma \left(\omega \right)$, and energy loss $L\left(\omega \right)$. These physical quantities are investigated in this section for pristine and F-doped rutile TiO_2_ to determine its optical properties. Our investigation focuses on the energy range 0–5 eV, which includes both visible and UV regions. To identify the visible and UV regions in the spectra, we can use the energy and wavelength relation, given by $E\left(\mathrm{eV}\right)=1240/\lambda (\mathrm{nm})$.

#### 3.5.1. Dielectric Functions

The dielectric function is a key quantity in studying the optical properties of materials, and it can be expressed in terms of its real and imaginary parts. The complex dielectric function is a crucial function in determining the optical properties of materials and is given by [50]

(3) $$\varepsilon \left(\omega \right)=i\varepsilon_{i}\left(\omega \right)+\varepsilon_{r}\left(\omega \right)$$

where $\varepsilon_{i}$ is an imaginary dielectric and $\varepsilon_{r}$ is a real dielectric function.

The real component of the dielectric function is used to determine the polarization and dispersion electromagnetic radiation in materials. The Kramers–Kronig relationship is used to compute the real component of dielectric and is given by the formula in Equation (4) [49], where p represents the primary value of the integral.

(4) $$\varepsilon_{r}\left(\omega \right)=1+2\left(\frac{p}{\pi }\right)\int_{0}^{\infty }\frac{\varepsilon_{i}(\omega \prime )\omega \prime }{\omega^{\prime 2}-\omega^{2}}$$

**Figure 10:**
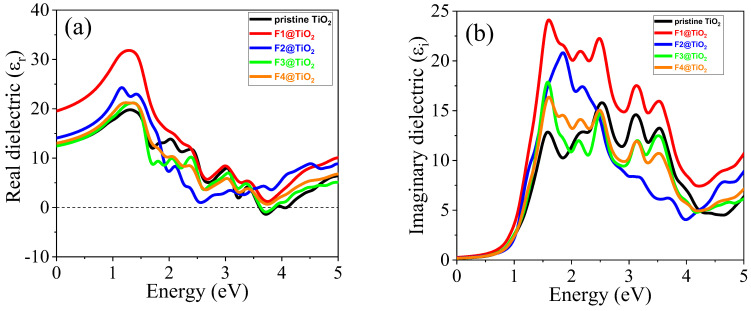
(**a**) Real dielectric function and (**b**) imaginary dielectric function of pure TiO_2_ and TiO_2_ doped with different non-metals.

The real dielectric function of pristine TiO_2_ and TiO_2_ doped with various F concentrations in the photon energy range 0–5 eV is shown in Figure 10a. The static values of the real part of the dielectric function ($\varepsilon_{r}\left(0\right))\;$of pure and doped rutile TiO_2_ at zero photon energy are given in Table 5. High values of ε_r_ usually correspond to low reflectivity and high transmissivity, meaning that the material is more effective at allowing light to be absorbed or pass through the surface. Among the doped materials, the F1-doped TiO_2_ showed the highest value, indicating the highest absorption characteristics of this doped material. In comparison with pristine TiO_2_, all doped materials possessed a higher value of real dielectric function in the energy range 0 to 1.70 eV, which confirms the enhanced absorption edges after doping with various concentrations. Negative values of the real part of the dielectric function are observed, while the photon energy increases to ultraviolet (UV), which indicates that the material exhibits high reflection of the incoming electromagnetic radiation and limited transmission. The positive values of the real dielectric function at static $\varepsilon_{r}\left(0\right)$ in visible light suggests that the materials exhibit low reflection, indicating favorable optical characteristics and transparency [51].

The imaginary part of the dielectric function is directly related to the material’s optical absorption and plays a significant role in characterizing how materials interact with electromagnetic radiation [52]. The imaginary component of the dielectric function is given by Equation (5) [49,53]:

(5) $$i\varepsilon_{i}=e^{2}\hbar^{2}\left(\frac{1}{\pi m^{2}\omega^{2}}\right)\sum_{V,C}{\left|\left\langle \varphi_{C}|\hat{e_{j}}.\vec{M}|\varphi_{V}\right\rangle \right|}^{2}\sigma (E_{C}-E_{V}-\hbar \omega )$$

where *m* and e are the mass and charge of an electron, respectively; ℏ is reduced Planck’s constant; ω is frequency; φ is wave function; C and V represent the conduction and valence band, respectively; $\hat{e_{j}}$ unit vector shows the direction of the electromagnetic radiation;$\;\vec{M}$ is the momentum operator; and $E_{C}$ and $E_{V}$ are the conduction and valence energy, respectively.

The computed imaginary part of the dielectric function for pristine and F-doped materials with various concentrations is shown in Figure 10b. The highest peak corresponds to energies where strong transitions occur between electronic states. Thus, it shows the transitions of electrons from the valence band states of O 2p to the unoccupied conduction band Ti 3d states. The transition from occupied states to unoccupied states is enhanced when F is incorporated into the lattice. F1-doped materials showed the highest peak, indicating strong transitions compared to other materials, which may be attributed to its smallest bandgap value. Thus, its greatest absorption indicates that this concentration-doped material is a promising candidate for optoelectronic applications. Furthermore, the presence of various peaks after F doping indicates the interband transitions of electrons from valence band to conduction band, which is crucial for photocatalysis to take place.

The dielectric function is used to calculate various optical properties, including refractive index (n), extinction coefficient (κ), optical conductivity (σ), electron energy loss (L), reflectivity (R), and absorption coefficients (α) using specific formulas [32,54,55]:

(6) $$n\left(\omega \right)=\sqrt{\frac{\sqrt{\varepsilon_{r}^{2}\left(\omega \right)+\varepsilon_{i}^{2}\left(\omega \right)}+\varepsilon_{r}(\omega )}{2}}$$

(7) $$\kappa \left(\omega \right)=\sqrt{\frac{\sqrt{\varepsilon_{r}^{2}\left(\omega \right)+\varepsilon_{i}^{2}\left(\omega \right)}-\varepsilon_{r}(\omega )}{2}}$$

(8) $$R(\omega )=\frac{1+\kappa^{2}-(2n-n^{2})}{1+\kappa^{2}-(-2n-n^{2})}$$

(9) $$L\left(\omega \right)=\frac{\varepsilon_{i}^{2}(\omega )}{\varepsilon_{i}^{2}\left(\omega \right)+\varepsilon_{r}^{2}\left(\omega \right)}$$

(10) $$\alpha \left(\omega \right)=\frac{4\pi \kappa \left(\omega \right)}{\lambda }$$

where c is the speed of light and λ is the wavelength.

(11) $$\sigma \left(\omega \right)=\frac{\alpha \left(\omega \right)n\left(\omega \right)c}{4\pi }$$

#### 3.5.2. Refractive Index (n)

**Figure 11:**
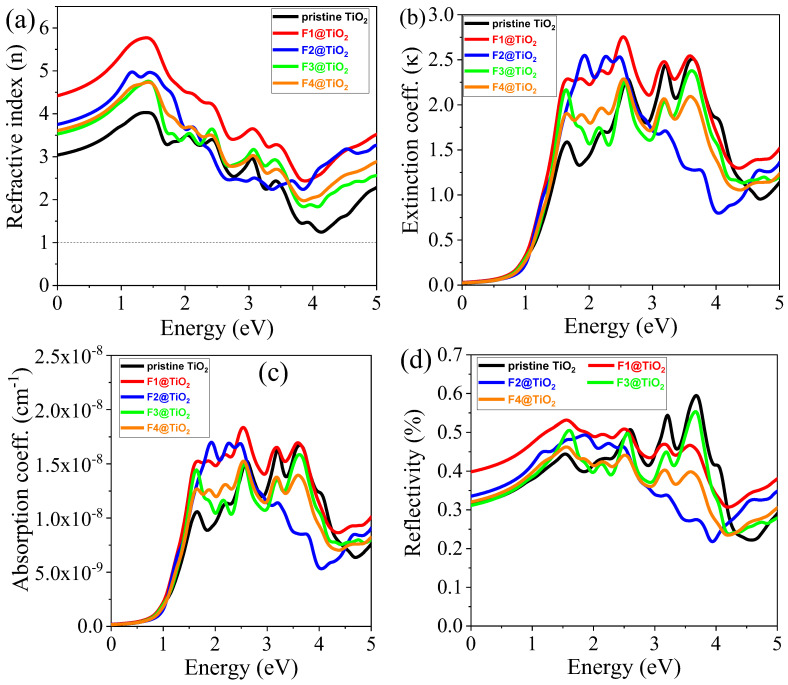
The calculated (**a**) $n\left(\omega \right)$ , (**b**) $\kappa \left(\omega \right)$, (**c**) $\alpha \left(\omega \right),$ and (**d**) R(ω) of pristine and F-doped rutile TiO_2_.

The study of the refractive index of rutile TiO_2_, particularly under various concentrations of F doping, provides valuable insights into its optical properties, which play a crucial role in photocatalysis. In the context of photocatalytic applications, the refractive index is a key factor influencing light interaction with the material, which directly impacts the material’s efficiency under visible light irradiation. The refractive properties of materials can be described by a real dielectric function. The refractive index of pristine and F-doped TiO_2_ in the photon energy range of 0–5 eV is shown in Figure 11a. The obtained static refractive index for pristine and rutile TiO_2_ doped with F1, F2, F3, and F4 are 3.01, 4.42, 3.75, 3.52, and 3.60, respectively, as shown in Table 5. The results show that F doping significantly influences the refractive index of TiO_2_. Pristine TiO_2_ exhibits a refractive index of 3.01, which increases as the doping concentration of F increases. The F1-doped rutile TiO_2_, with the largest refractive index of 4.42, stands out among all other samples. This enhancement is attributed to the reduction in the bandgap, which allows the material to absorb light more efficiently in the visible spectrum, thus improving its suitability for photocatalytic applications. The lower bandgap in F-doped TiO_2_ creates an enhanced light absorption capacity, which is a critical factor for increasing photocatalytic activity under visible light.

A higher refractive index indicates that the material is optically denser, meaning that it can bend and slow down light more effectively than materials with a lower refractive index [56,57]. This property is advantageous for photocatalytic applications because it allows for greater light–matter interaction, enhancing the efficiency of photocatalytic reactions. The denser optical characteristics of F-doped TiO_2_ materials can lead to more efficient absorption of photons, which are crucial for initiating photocatalytic reactions, especially under visible light irradiation. Moreover, the high refractive index suggests that these materials can be useful in solar cell applications, where efficient light management is required. The trends observed in the refractive index suggest that increasing the F doping concentration not only improves the optical properties but also may help in optimizing the photocatalytic efficiency by allowing for better control over light absorption and energy conversion. In applications like water splitting, organic pollutant degradation, or CO_2_ reduction, enhancing light absorption and improving the rate of electron–hole pair generation are critical factors for achieving high photocatalytic performance.

#### 3.5.3. Extinction Coefficient (κ)

The extinction coefficient (κ) is a critical optical property that characterizes how much light is absorbed or scattered as it passes through a material, directly influencing the material’s performance in applications including photocatalysis and photovoltaics. The calculated extinction coefficients for pristine and F-doped TiO_2_ is shown in Figure 11b, which provides valuable insight into the impact of F doping on the material’s light absorption properties. The results reveal that F doping enhances the extinction coefficient at lower photon energies, with doped TiO_2_ materials showing higher peaks compared to pristine TiO_2_. This is likely due to the bandgap narrowing and the introduction of defects or new energy states associated with the dopant. The shift in the absorption edge towards the visible region for the doped materials indicates that these modifications enhance the material’s ability to absorb visible light, which is a key requirement for efficient photocatalytic activity under visible light irradiation. In photocatalytic applications, the ability to absorb light efficiently, particularly in the visible region, is essential for driving reactions such as water splitting and pollutant degradation. The increased extinction coefficient in F-doped TiO_2_ suggests that these materials, particularly those with optimal doping concentrations, are better suited for photocatalytic processes under visible light. This makes F-doped TiO_2_ a promising candidate for solar-driven photocatalysis applications, where efficient light utilization is crucial for electron–hole generation.

#### 3.5.4. Absorption Coefficient (α)

The absorption coefficient (α) is a fundamental property in understanding how materials interact with light, making it essential for applications in photocatalysis, photovoltaics, and optoelectronics. The absorption coefficient directly influences how much light a material can absorb at different wavelengths, which in turn determines its ability to generate charge carriers that drive photocatalytic reactions. The calculated absorption coefficient of pristine TiO_2_ and TiO_2_ doped with various F concentrations in the photon energy range from 0 to 5 eV is depicted in Figure 11c. The results demonstrate that F doping significantly affects the absorption characteristics of TiO_2_, particularly in the visible light region. The presence of F as a dopant enhances the light absorption ability of TiO_2_, especially under visible light irradiation. This enhancement is highly beneficial for photocatalysis applications, which often rely on the material’s ability to efficiently absorb visible light to drive photoreactions. In the visible region, when the concentration of F increases, the absorption coefficient decreases. Despite this, the F1-doped TiO_2_ exhibits the highest absorption coefficient compared to the other doping concentrations. This observation indicates that the F1-doped TiO_2_ is the most efficient material in terms of light absorption across a wide range of wavelengths. The optimized light absorption ability of the F1-doped material suggests that it is well suited for photocatalytic applications, as it can absorb a significant portion of the solar spectrum, especially the visible light portion, which is essential for maximizing photocatalytic efficiency.

The enhanced photocatalytic performance observed at lower F concentrations can be attributed to the introduction of a small number of defects that effectively narrow the bandgap of TiO_2_. This bandgap narrowing allows for better absorption of visible light, thereby improving photocatalytic efficiency. However, as the F concentration increases, excessive defect states are introduced into the material. These defects can act as recombination centers for photogenerated charge carriers, leading to an increase in the recombination rate. Higher recombination rates reduce the number of charge carriers available for photocatalytic reactions, thereby decreasing the overall photocatalytic performance. Thus, while low levels of doping enhance photocatalytic efficiency by optimizing light absorption, higher doping concentrations can have a detrimental effect due to the increased recombination of charge carriers. A similar experimental study reported enhanced photocatalytic performance in Se-doped TiO_2_ due to the narrower bandgap [58]. In this study, Se concentrations varied up to 17.1 at.%, with the 13.63 at.% concentration showing the most significant improvement in the photocatalytic activity of TiO_2_. Additionally, the study found that lower Se concentrations slightly improved the TiO_2_ crystalline structure, while higher Se concentrations led to distortions in the crystalline structure.

#### 3.5.5. Reflectivity (R)

Reflectivity can indicate changes in surface states, bandgap modification, and the efficiency of light absorption, which is relevant for applications like photovoltaic or photocatalysis. The calculated reflectivity of pristine and F-doped TiO_2_ is shown in Figure 11d. The static reflectivity of pristine, F1-doped, F2-doped, F3-doped, and F4-doped TiO_2_ is 0.33, 0.39, 0.34, 0.31, and 0.32, respectively, as shown in Table 5. The doped materials with high reflectivity can be used for high-reflect coating applications [59]. Reflectivity of all materials increased to 1.60 eV and then randomly decreased to 2.44 eV. For further increase in photon energy from 2.95 eV to 3.70 eV, the reflectivity of the materials randomly increased.

#### 3.5.6. Electron Energy Loss Spectrum (EELS)

**Figure 12:**
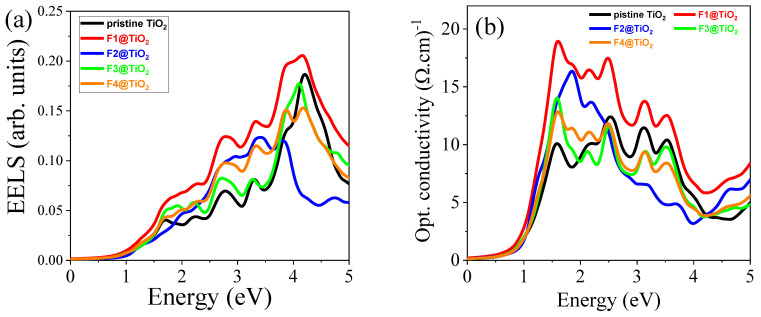
The computed (**a**) $L\left(\omega \right)$ and (**b**) $\sigma \left(\omega \right)$ of pristine and F-doped rutile TiO_2_.

The electron energy loss spectrum (EELS) provides valuable insight into the interactions between electrons and materials, offering key information about a material’s electronic structure and its potential for photocatalytic applications. The EELS of pristine TiO_2_ and TiO_2_ with various F concentrations of doping is depicted in Figure 12a. It shows that the EELS for pristine and F-doped TiO_2_ reveals important trends that can inform the material’s photocatalytic efficiency under visible light. In the low-energy region below 1 eV, there is minimal electron energy loss for all materials except for the F1-doped TiO_2_, exhibiting a small energy loss. This suggests that F doping, particularly at the F1 concentration, introduces subtle changes to the electronic structure of rutile TiO_2_, which potentially improves its interaction with light and electrons. Between 1 eV and 4 eV, the energy loss increases for all materials, with doped TiO_2_ exhibiting more significant losses compared to pristine TiO_2_. This indicates that F doping enhances the interaction between electrons and the material, which is beneficial for photocatalysis as greater electron mobility and interaction are key factors for improving photocatalytic reactions. Among all doped materials, the F1-doped material has the highest energy loss, as shown in Table 5, which may be attributed to the composition elements and electronic structures of this material. In the higher photon energy region, the EELS increased, which can be attributed to less interaction of electrons with materials.

#### 3.5.7. Optical Conductivity (σ)

Optical conductivity is a critical factor in determining the photocatalytic performance of semiconductor materials, as it is directly related to the availability of photogenerated charge carriers for photocatalytic reactions. The calculated conductivity of pristine and F-doped materials is shown in Figure 12b. The doped materials have higher conductivity than undoped TiO_2_, as shown in Table 5. The F doping in TiO_2_ significantly enhances the optical conductivity compared to pristine TiO_2_. Thus, doping introduces defects in the crystal lattice, which facilitates the presence of photogenerated charge carriers. This is beneficial for photocatalysis, as efficient charge carrier generation and mobility are essential for driving photocatalytic reactions. Comparing doped materials, when the F doping concentration increases, optical conductivity decreases. This is attributed to the widening of the bandgap when the F concentration increases. While this may limit the materials’ performance at higher doping concentrations, the optimized F1 doping that balances conductivity and bandgap narrowing can enhance photocatalytic efficiency by promoting efficient charge separation and transfer under visible light irradiation.

### 3.6. Photocatalytic Activity

Photocatalysis is a process that uses light energy (photon) and a semiconductor as a catalyst to drive chemical reactions. The photocatalytic performance of pristine rutile TiO_2_ and TiO_2_ doped with various F concentrations can be evaluated by calculating the valence band maximum (VBM) and conduction band minimum (CBM) relative to the normal hydrogen electrode (NHE). This can be achieved using the following empirical formulas [60]. The valence band edge ($E_{VB}$) is expressed as

(12) $$E_{VB}=\chi -E_{e}+\frac{1}{2}E_{g}$$

The conduction band edge ($E_{CB}$) is defined as

(13) $$E_{CB}=E_{VB}-E_{g}$$

In these equations, χ represents the electronegativity of TiO_2_ (5.81 eV), $E_{e}$ indicates the electron free energy on the hydrogen scale (4.5 eV), and $E_{g}$ is the energy bandgap of pristine and doped TiO_2_. Thus, using the calculated bandgap, it is possible to determine the values of $E_{VB}$ and $E_{CB}$ for pristine and doped TiO_2_.

**Figure 13:**
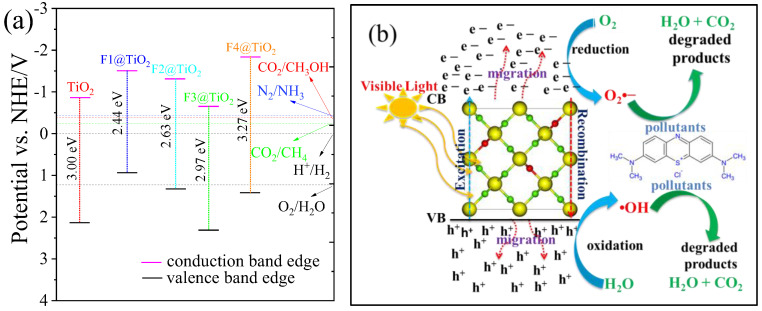
(**a**) Band edge positions of pristine TiO_2_ and rutile TiO_2_ doped with various concentrations, and (**b**) the possible photocatalysis mechanism of pollutant degradation.

The computed band edge positions of pristine and doped TiO_2_ is depicted in Figure 13a. The edges refer to the energy requirements for the electron or hole to move to a state where it can participate in the desired chemical transformation. These values are often associated with redox potentials, which indicate the energy required to reduce or oxidize a substance under specific conditions. Photocatalytic water splitting involves the use of light energy to split H_2_O into H_2_ and O_2_, as shown in Equation (14). In this case, the CBM of the photocatalyst must be more negative than the reduction potential for hydrogen ion reduction, which is 0 V (vs. standard hydrogen electrode, SHE,) under standard conditions (pH 0), whereas the VBM must be more positive than the oxidation potential of water to produce O_2_, which is +1.23 V (vs. NHE) under standard conditions [61]. As shown in Figure 13a, the CMB of pristine and all concentrations of doped TiO_2_ are more negative. For the VBM, except the 0.0625-doped TiO_2_, all systems are more positive, which indicates that these materials are suitable for H_2_ production except the 0.0625-doped TiO_2_.

(14) $$2H_{2}O+light\to 2H_{2}+O_{2}$$

The semiconductor absorbs photons, generating electrons and holes, as shown in Figure 11b. The electrons reduce H^+^ from H_2_O, producing H_2_, while the holes oxidize water to form O_2_. This mechanism is used to produce H_2_ as a clean fuel.

The photocatalytic process in the reduction of CO_2_ to methane (CH_4_) using light energy can contribute to carbon recycling and provide a renewable source of methane for energy. The relevant reaction is

(15) $$CO_{2}+4H_{2}+light\to {CH}_{4}+2H_{2}O$$

For CO_2_ reduction to CH_4_, the CBM must have sufficient negative potential to reduce CO_2_ to a carbon-based species. The reduction potential of CO_2_ to CH_4_ is about −0.24 V (vs. NHE) under standard conditions. Thus, the CBM of the photocatalyst should be more negative than −0.24 V (vs. NHE). The VBM must be positive enough to oxidize water to O_2_, which requires a VBM above +1.23 V (vs. NHE). As can be seen from Figure 13a, the VBM and CBM of all materials except the 0.0625-doped TiO_2_ are suitable for CO_2_ reduction to CH_4_ for energy.

In photocatalytic ammonia synthesis, N_2_ is reduced to ammonia (NH_3_) under light exposure. For the reduction of N_2_ to NH_3_, the CBM should be more negative than −0.43 V (vs. NHE). As with other photocatalytic reactions, the VBM must be above +1.23 V (vs. NHE) for water oxidation to generate oxygen.

(16) $$N_{2}+3H_{2}+light\to 2NH_{3}$$

Photocatalytic reduction of CO_2_ to methanol (CH_3_OH) is an important process for carbon recycling. Methanol is a valuable chemical and fuel, and photocatalytic CO_2_ reduction provides a potential pathway to mitigate CO_2_ emissions. The reduction potential for CO_2_ to CH_3_OH is approximately −0.38 V (vs. NHE). Therefore, the CBM must be more negative than −0.38 V (vs. NHE). As usual, the VBM must be more positive than +1.23 V (vs. NHE) to oxidize water to oxygen.

(17) $$CO_{2}+3H_{2}+light\to CH_{3}OH+H_{2}O$$

#### Mechanism of Photocatalytic Wastewater Treatment

The basic mechanism behind photocatalytic wastewater treatment involves the use of a semiconductor material as a photocatalyst. When the photocatalyst is illuminated by light, it generates electron–hole pairs, which can then interact with the contaminants in the wastewater. The general mechanism of photocatalytic wastewater treatment is shown in Figure 13b. When the light is irradiated on photocatalysts (either pristine or F-doped TiO_2_), electron–hole pairs are generated, as given in Equation (18). Then, the photogenerated charge carriers migrate to the surface of the photocatalysts.

(18) $$Fdoped\;TiO_{2}+h\nu \to e^{-}+h^{+}$$

where hν is the energy from light, $e^{-}$ is the electron in the conduction band, and $h^{+}$ is the hole in the valence band.

The electrons in the conduction band ($e^{-}$) can reduce oxygen molecules to generate superoxide anions (O_2_•−).

(19) $$O_{2}+e^{-}\to O_{2}•-$$

The holes in the valence band ($h^{+}$) can oxidize water molecules to produce hydroxyl radicals (•OH).

(20) $$H_{2}O+h^{+}\to •\mathrm{OH}+H^{+}$$

The hydroxyl radicals (•OH) and superoxide anions (O_2_^•^−) attack organic molecules like dyes, pharmaceuticals, and pesticides in the wastewater, breaking them down into smaller, less harmful molecules, such as CO_2_ and H_2_O.

### 3.7. Experimental Recommendation

Further experimental validation of these theoretical predictions is essential to confirm the practical advantages of these materials in real-world photocatalytic systems. To synthesize TiO_2_ with varying F concentrations, both bottom-up and top-down approaches can be employed. Among the bottom-up methods, chemical solution techniques, such as co-precipitation, combustion, sol–gel, and hydrothermal synthesis, are particularly promising [61]. These methods are advantageous due to their relatively simple synthesis procedures, cost-effectiveness, and the ability to easily control the phase and particle size of the resulting F-doped TiO_2_ photocatalyst.

Furthermore, as shown in Table 6, several experimental studies using various techniques have investigated F- and other non-metal-doped rutile TiO_2_. A common observation across these studies is a reduction in bandgap energy upon doping, which consequently enhances photocatalytic activity. This enhancement is typically attributed to the red shift of the absorption edge, allowing the doped TiO_2_ to absorb a broader spectrum of visible light. Our theoretical calculations are in strong agreement with these findings. Specifically, we observe a consistent bandgap narrowing across all doping concentrations, except at the highest concentration of 0.25, where the trend deviates slightly. This suggests a possible saturation or structural distortion effect at higher dopant levels, which has also been occasionally noted in the experimental literature.

Moreover, the experimental data highlight that improved photocatalytic performance is not solely dependent on bandgap narrowing. In some instances, even when the bandgap remains constant or slightly increases, enhanced activity is still reported. This is often attributed to improved charge separation dynamics, where dopants act as electrons or hole traps, thereby reducing the recombination rate of photogenerated carriers. Several studies listed in Table 6 corroborate this mechanism, indicating increased degradation efficiencies (represented by rate constant *k* and %), even without significant bandgap shifts. Therefore, our theoretical results are well supported by the experimental findings, in terms of both bandgap modulation and the expected enhancement in photocatalytic activity.

## 4. Conclusions

In this study, a DFT + U approach was used to investigate the effects of fluorine (F) doping on rutile TiO_2_ employing the Quantum ESPRESSO code. Structural analysis revealed that the lattice constants of pure rutile TiO_2_ were *a* = *b* = 4.61 Å and *c* = 2.97 Å, and that these values increased upon F doping. This is attributed to the slight difference in atomic radii between the F and O atoms. Additionally, the bond lengths in F-doped rutile TiO_2_ were longer than in pristine rutile TiO_2_, indicating lattice distortion caused by the substitution of F at oxygen sites. Ab initio molecular dynamics simulations conducted at 1000 K demonstrate that both pristine and F-doped rutile TiO_2_ retain their structural integrity, confirming their thermal stability and suitability for photocatalytic applications under high-temperature conditions. The bandgap of pristine rutile TiO_2_ was calculated to be 3.0 eV, which aligns well with the experimental data. For F-doped TiO_2_, the bandgap decreased to 2.44 eV for 0.0625 doping, 2.63 eV for 0.125 doping, and 2.97 eV for 0.1875 doping, and increased to 3.26 eV for 0.25 doping. Partial density of states (PDOS) analysis showed that the valence band was predominantly composed of O 2p orbitals, while the conduction band was mainly composed of Ti 3d orbitals. The F 2p orbitals contributed more to the valence band, confirming the successful substitution of F at the oxygen sites. The imaginary part of the dielectric function indicated that the absorption edges of TiO_2_ doped with 0.0625, 0.125, and 0.1875 F atoms shifted toward the visible region, suggesting improved photocatalytic activity. Additional optical properties, such as refractive index, extinction coefficient, reflectivity, electron energy loss spectrum, and optical conductivity, further confirmed the enhanced visible-light response of TiO_2_ doped with 0.0625, 0.125, and 0.25 F. Overall, this work demonstrates that controlled F doping serves as a powerful strategy to tailor the intrinsic properties of rutile TiO_2_, enabling effective bandgap engineering and improved visible-light absorption. By offering predictive insights into the influence of F doping, our findings provide a valuable theoretical foundation for guiding experimental synthesis and optimizing dopant concentrations in photocatalytic material design. This approach aligns with broader efforts in rational materials engineering and may stimulate further exploration of halogen-doped oxide semiconductors for energy and environmental applications.

## Figures and Tables

**Table 1:** The obtained lattice constants of pure rutile TiO_2_ and TiO_2_ doped with various F concentrations.

Materials		Pure TiO_2_	F1@TiO_2_	F2@TiO_2_	F3@TiO_2_	F4@TiO_2_
Lattice constants (Å) Present work	a = b	4.610	4.626	4.639	4.657	4.671
	c	2.975	3.012	3.042	3.074	3.105
Lattice constants (Å) Other DFT work	a = b	4.637 ^a^	--	--	--	--
	c	2.989 ^a^	--	--	--	--
Experiment	a = b	4.600 ^b^	--	--	--	--
	c	2.959 ^b^	--	--	--	--
Volume (Å)^3^		62.91	64.15	64.87	65.38	66.46

Other values are taken from Refs ^a^ [36] and ^b^ [37].

**Table 2:** The obtained bond angles, bond length, and formation energies of pure rutile TiO_2_ and TiO_2_ doped with various F concentrations.

Compounds	Bond Length (Å)	Bond Angle (°)	Formation Energy (eV)
Pure TiO_2_	Ti-O = 1.9518	O-Ti-O = 90	
F1@TiO_2_	Ti-O = 1.9520 Ti-F = 1.9799	O-Ti-O = 90 F-Ti-O = 89.91	−2.73
F2@TiO_2_	Ti-O = 1.9522 Ti-F = 2.0026	O-Ti-O = 90 F-Ti-O = 88.1	−2.91
F3@TiO_2_	Ti-O = 1.9527 Ti-F = 2.0036	O-Ti-O = 90 F-Ti-O = 85.91	−3.35
F4@TiO_2_	Ti-O = 1.9530 Ti-F = 2.0062	O-Ti-O = 90 F-Ti-O = 81.16	−3.68

**Table 5:** The maximum electron energy loss $L_{max}\left(\omega \right)$ , absorption coefficient $\alpha_{max}\left(\omega \right)$, optical conductivity $\sigma_{max}\left(\omega \right),$ and real dielectric $\varepsilon_{r}\left(0\right)$, refractive index $n\left(0\right)$, and reflectance R(0) at zero energy.

Material	$\varepsilon_{r}\left(0\right)$	$n\left(0\right)$	R(0)	$L_{max}\left(\omega \right)$	$\alpha_{max}\left(\omega \right)$ $\left(10^{-8}/\mathrm{cm}\right)$	$\sigma_{max}\left(\omega \right)$ ${\left(Ω.\mathrm{cm}\right)}^{-1}$
Pure TiO_2_	12.54	3.01	0.33	0.19	1.62	12.40
F1@TiO_2_	19.55	4.42	0.39	0.21	1.84	18.95
F2@TiO_2_	14.08	3.75	0.34	0.12	1.70	16.36
F3@TiO_2_	12.46	3.52	0.31	0.18	1.53	14.04
F4@TiO_2_	13.03	3.60	0.32	0.15	1.51	12.86

**Table 6:** The experimental reports on photocatalytic activity of F- and other non-metal-doped rutile TiO_2_.

Material	Experimental Method	Bandgap (eV)	Light Irradiated	Pollutants	Degradation Efficiency	Ref.
F-doped TiO_2_	Sol–gel	3.19	Visible light	Methylene blue	K = 0.00542	[20]
Ag/F-TiO_2_	Sol–gel	2.35	Visible light	Methylene blue	K = 0.0189	[20]
Pure TiO_2_	Sol–gel	3.07	UV-vis	Acetone	Lower	[21]
F-doped TiO_2_	Sol–gel	3.03	UV-vis	Acetone	Higher	[21]
Pure TiO_2_	One-step synthesis	3.0	Visible light	Bisphenol A	Lower than doped	[22]
F-doped TiO_2_	One-step synthesis	1.5	Visible light	Bisphenol A	98%	[22]
Pure TiO_2_	Solid state reaction	3.09	UV	Methyl orange	K = 0.0042	[62]
F-doped TiO_2_	Solid state reaction	2.85	UV	Methyl orange	K = 0.0433	[62]
Pure TiO_2_	Sol–gel	2.96	Visible light	Methylene blue	Lower	[63]
C-doped TiO_2_	Sol–gel	2.37	Visible light	Methylene blue	K = 0.04513	[63]
Pure TiO_2_	Sol–gel	3.01	Visible light	Bisphenol A	K = 1.61	[64]
I-doped TiO_2_	Sol–gel	3.04	Visible light	Bisphenol A	K = 5.11	[64]
B-doped TiO_2_	Co-precipitation	2.85	Visible light	Rhodamine B	Enhanced	[65]
B-doped TiO_2_	One-step calcination	−	UV-visible	Atrazine	95%	[66]
Pure TiO_2_	Decomposition	3.1	Visible light	Rhodamine B	15%	[67]
N-doped TiO_2_	Decomposition	2.85	Visible light	Rhodamine B	90.3%	[67]
S-doped TiO_2_	Anodic oxidation	−	Visible light	Methylene blue	Enhanced	[68]

## Data Availability

Based on the reasonable request, the data that support the findings of this manuscript will be available from the corresponding author.
